# Dr. Walshman

**Published:** 1836-07

**Authors:** 


### DR. WALSHMAN.

APRIL 3d, 1836, at the advanced age of ninety, Dr. Walshman: he had been in practice during the last sixty years in the neighbourhood of Kennington, Clapham, Brixton, and Camberwell, where his experience and skill, more especially in Midwifery cases, made him a great authority. He belonged to the sect of Westleyan methodists, and was a great promoter of educational and other institutions connected with that respectable body. He has left property, it is said, to the amount of £200,000.

### BOOKS RECEIVED FOR REVIEW.

